# Distinct domains: a call for nuance in the categorization and evaluation of “Arts and Humanities” disciplines

**DOI:** 10.3389/frma.2025.1661966

**Published:** 2025-08-29

**Authors:** William E. Savage, Richard Wheeler, Anthony J. Olejniczak

**Affiliations:** Academic Analytics Research Center (AARC), Columbus, OH, United States

**Keywords:** research evaluation, bibliometrics, humanities, fine and performing arts, academic disciplines

## Introduction

Research universities frequently group the visual and performing arts with the lettered humanities into a single administrative unit^1^. Other well-known data sources in higher education use an identical approach to group these fields of study, including the US National Science Foundation's Survey of Earned Doctorates (NCSES, 2024). Moreover, “Arts and Humanities” is a subject category in Elsevier's All Science Journal Classification (ASJC), which in turn is used to classify journals in the Scopus bibliometric database and as a subject classification scheme for QS Rankings. Other influential rankings also group arts and humanities, including those from both US News and World Report and Times Higher Ed. Finally, many bibliometric studies published in scholarly journals also treat the arts and humanities disciplines as a single category (e.g., Fanelli and Glänzel, 2013; Golosovsky and Larivière, 2021; Thelwall and Maflahi, 2022). Taken together, these examples demonstrate how routinely the arts and humanities are combined and evaluated as a single disciplinary category. Unfortunately, the ready availability of data in this combined format, in concert with ranking schemes that measure this grouping as a single entity, may encourage research administrators to treat these domains as unitary in their assessments, strategy, and planning.

In this opinion, we argue that this practice is not only imprecise but is fundamentally inequitable and distorts the scholarly contributions of both domains. Drawing on a 10-year tranche of publication data, we demonstrate a stark divergence in the tempo and modality of scholarly output between the “Visual and Performing Arts” and “Humanities” groups of disciplines. Failure to recognize this distinction disadvantages arts faculty in assessments, deflates the perceived productivity of humanities units, and can lead to flawed strategic decision-making by academic leaders. Our argument is grounded in the broader recognition of a need for discipline-sensitive evaluation, a principle supported by the Leiden Manifesto for Research Metrics (Hicks et al., 2015) which advises that “Quantitative evaluation should support qualitative, expert assessment” and that performance be measured “against the research missions of the institution, group or researcher.” Furthermore, this analysis builds on our previous work establishing significant differences inscholarly rhythms across all major academic fields, from the rapid, collaborative publishing cycles in STEM to the long-term, monograph-focused work in the humanities (Olejniczak et al., 2021b). The present opinion moves beyond those broad patterns to scrutinize the specific, problematic aggregation of visual and performing arts and humanities disciplines, and advocates for a more nuanced approach.

## Different domains, different publication patterns

### Our classification of disciplines

The humanities and visual and performing arts are similar in that they both explore the human experience, both foster critical thinking, creativity, and empathy, and both seek to interpret and understand human culture. These domains differ, however, in the methods they employ to demonstrate their scholarly and artistic productivity. Humanities methodologies focus primarily on ways to analyze and interpret human culture, events, and artifacts. Scholarly communication and the dissemination of knowledge is largely through the written word. Visual and performing arts inquiry, in general, focuses on the process of artistic creation and expressive acts that produce artifacts. For the remainder of this opinion, we define “humanities” to include disciplines whose primary means of knowledge dissemination is the written word, such as History, Philosophy, Language and Literature studies, and Area, Ethnic, and Gender studies. We define “Visual and Performing Arts” to include disciplines whose primary forms of productivity are centered on performance, composition, and exhibition. Visual and Performing Arts disciplines include, e.g., Dance, Music, Theater, Painting, and Sculpture. We acknowledge, of course, that many scholars in the visual and performing arts disseminate their work through the written word, publishing histories, criticism, and other texts. In bibliometric databases, it is likely the works of this group whose works are classified as “Arts and Humanities.”

### Publishing rhythms

We analyzed a dataset previously made public (Olejniczak et al., 2021a) that includes publication rates over various timeframes for scholars by academic discipline. In a typical humanities discipline, ~65% of scholars have published at least one article within a 3-year period (range: 46–81%). This rate rises to ~85% of scholars having published an article within a 10-year period (range: 61–93%). In stark contrast, disciplines in the visual and performing arts exhibit faculty article publication rates of only 21% at the 3-year mark (range: 21–25%) and reaching just 32% after a full decade (range: 30–34%)^2^. Many, probably most, of these scholarly articles are produced by faculty housed in visual and performing arts units but who do critical or historical interpretive work that resembles the work of humanists—for example, theater historians. Although visual and performing arts units often include faculty who publish scholarly articles, such publications do not provide a useful indicator of the overall productivity in the discipline.

Two recent studies confirm the slow publishing rhythms of visual and performing arts disciplines. A bibliometric study of the Scopus database searching for articles published on “visual arts” between the years 1952 and 2020 found only 1,727 articles, 1,173 articles of which (68%) were published in the 10 years between 2011 and 2020 (González-Zamar and Abad-Segura, 2021). Another bibliometric analysis focused on the categories “visual arts” and “performing arts” indexed in the Scopus database between 2016 and 2020 (Agámez-Llanos et al., 2023). That study found 1,342 articles. Of all authors of those articles, 97% published only a single article over the 5 years analyzed. Sixteen authors had published two articles, and four authors had published three articles.

### Visual and performing arts scholarship and the bibliometric archive

The distinct publishing rhythms of the humanities and the visual and performing arts reflect fundamental differences in their disciplinary activities. Much of the scholarly labor in the arts is practice-based: practitioners compose music, choreograph dances, and create or curate art for exhibition. Furthermore, even when arts scholars focus on the written word, publishing criticism, analysis, and histories, their publication rhythms often differ vastly from those in the humanities. This combination of creative work and distinct publication cycles means the full scope of scholarship in the visual and performing arts is not adequately captured by periodical literature or indexed in bibliometric databases. Researchers in the visual and performing arts point out that any attempt at evaluation, especially using standards typically set in STEM fields (which generally rely heavily on bibliometric indicators), provides an incomplete assessment and does a disservice to arts faculty. Bibliometrics cannot convey the full significance of performing and visual arts outputs (Miskey and Saladino, 2023; Thelwall and Delgado, 2015; Zhao and Minns, 2019). Simply put, the artifacts generated by visual and performing arts faculty are unlikely to appear in bibliometric archives.

## Discussion

The singular “Arts and Humanities” category that is so often encountered in data sources and university ranking schemes creates significant distortions that disadvantage both humanities scholars and visual and performing arts scholars. When the slower publication tempo of the visual and performing arts faculty who disseminate their work through textual publications is included in divisional assessments, aggregate productivity metrics of the division (e.g., articles per faculty member) appear less active than it is. The greater the size of the visual and performing arts faculty relative to the humanities faculty, the more likely a university will be penalized in ranking schemes through lower divisional per capita publication and citation counts. Perhaps more critically, evaluating performing and visual arts units within a “lettered humanities” context imposes an inappropriate standard. This framework is flawed on two fronts. First, it judges many scholars not by their primary modes of knowledge generation (juried exhibitions, musical compositions, theatrical performances) but by textual publications. Second, for arts scholars who do focus on publishing, it applies a metric (the journal article) that operates on a vastly different production rhythm than in the humanities, making direct comparisons misleading. For the purposes of equitable and meaningful evaluation, these distinct academic domains must be analyzed independently. The scholarly contributions of faculty in the visual and performing arts should be assessed within the context of their peer disciplines rather than being measured against the publication norms of adjacent but fundamentally different fields.

The use of metric indicators to guide university research strategy and assess research impact is now widespread. These indicators are often based on some aspect of scholarly communication through the publication of journal articles or other written artifacts. University strategy and planning exercises that rely on these data have real-world implications for individuals and academic units alike, including helping to decide who to hire, promote, or retain, how to allocate faculty lines, in which areas of investigation to invest resources, and many more. The common practice of bundling the visual and performing arts with the humanities creates a distorted and inequitable picture of scholarship. We believe the curators of influential database sources, including those that aggregate scholarly publications as well as the architects of global ranking systems, should take the critical step of disaggregating the “Arts and Humanities” category. By presenting these domains separately, they would not only provide more accurate data but also fulfill their responsibility to enable, rather than obstruct, fair assessment.

Concurrently, we urge university research administrators, institutional analysts, and faculty members to demand this nuance from their data providers and, in its absence, to perform this crucial separation themselves and include additional qualitative indicators, especially for the visual and performing arts. Evaluating artistic creation, criticism, history, or analysis through the same lens that is applied to the humanities represents a fundamental misunderstanding, and it devalues the core productivity of an entire domain. Nuanced representations of disciplinarily distinct activities are essential for equitable assessment, sound strategy, and intellectual honesty.

## Footnotes

^1^Examples include Harvard University's Arts and Humanities Division; the University of Notre Dame's College of Arts and Letters; and the University of Cambridge's School of Arts and Humanities.

^2^The publication patterns observed for the discipline “Art History and Criticism” occupy a middle-ground between the otherwise distinct humanities and visual and performing arts categories.
